# Genetic Polymorphisms of XRCC1 and Leukemia Risk: A Meta-Analysis of 19 Case-Control Studies

**DOI:** 10.1371/journal.pone.0080687

**Published:** 2013-11-25

**Authors:** Haijun Zhang, Hang Liu, Gaofeng Jiang

**Affiliations:** 1 Department of Cell and Developmental Biology, Weill Medical College of Cornell University, New York, New York, United States of America; 2 Columbia University School of Social Work, New York, New York, United States of America; 3 School of Public Health, Medical College, Wuhan University of Science and Technology, Wuhan, Hubei, China; Nanjing Medical University, China

## Abstract

**Objective:**

Three common X-ray repair cross-complementing groups 1 (XRCC1) polymorphisms, Arg399Gln, Arg194Trp, and Arg280His, have been reported to be implicated in the development of leukemia. However, previous results from different studies were inconsistent. Consequently, we performed a meta-analysis in order to accurately evaluate the association between XRCC1 Arg399Gln, Arg194Trp, and Arg280His polymorphisms and leukemia risk.

**Methods:**

Through computerized searching of PubMed, ISI Web of Knowledge, Cochrane, EBSCO, and OpenGrey databases, and manually searching relevant references, a total of 19 studies with 3387 cases and 6168 controls for Arg399Gln (G>A) polymorphism, 12 studies with 2043 cases and 4550 controls for Arg194Trp (C>T), and 6 studies with 1445 cases and 1905 controls for Arg280His (G>A) were collected to perform meta-analysis and stratified analysis to explore the associations between these variants and leukemia susceptibility. Based on three genetic models, the codominant model, dominant model and recessive model, odds ratios (ORs) as well as their 95% confidence intervals (CIs) were used to evaluate the association strength between XRCC1 genotypes and leukemia risk.

**Results:**

With respect to overall leukemia susceptibility, no association was detected. In stratified analyses by tumor type, Arg399Gln was associated with higher acute lymphoblastic leukemia (ALL) risk (AA vs. GG, OR  =  1.50, 95% CI: 1.11-2.02; AA+GA vs. GG, OR  =  1.35, 95% CI: 1.02-1.78). Additionally, Arg399Gln, Arg194Trp, and Arg280His may influence the susceptibilities of some leukemia type and race populations.

**Conclusion:**

This meta-analysis indicates these three polymorphisms of XRCC1 do not associate with overall leukemia risks but could be associated with the risks for some specific subgroups.

## Introduction

Leukemia is one of the most common human cancers, with an estimated 48610 new cases and 23720 deaths expected in the US in 2013 [1]. According to the cell type and growth rate, leukemia can be classified into four groups: acute myeloid leukemia (AML), acute lymphocytic leukemia (ALL), chronic myeloid leukemia (CML), and chronic lymphocytic leukemia (CLL). Although studies for leukemogenesis have been conducted for many years, the mechanisms underlying the development of this hemotologic malignancy remains unclear.

Impaired DNA repair may be associated with increased susceptibility to human cancers [2]. X-ray repair cross-complementing groups 1 (XRCC1) binds to DNA repair related proteins and takes part in the DNA repair process [3], [4], [5]. In the past decade, a number of studies have been performed to explore the relationship between three common XRCC1 single nucleotide polymorphisms (SNPs)—Arg399Gln (base G to A polymorphism), Arg194Trp (base C to T polymorphism), and Arg280His (base G to A polymorphism)—and leukemia risk. However, the conclusions of these studies are inconsistent. Therefore, a meta-analysis followed by stratified analysis of 19 published studies was performed to estimate the association between XRCC1 Arg399Gln, Arg194Trp, and Arg280His polymorphisms and leukemia risk.

## Materials and Methods

### Study identification

Computer bibliographic searches through PubMed, ISI Web of Knowledge, Cochrane, EBSCO, and grey literature database OpenGrey were conducted using the keywords: “leukemia,” “leukaemia” and “polymorphisms,” “genotypes,” “variants,” and “XRCC1,” “X-ray repair cross-complementing groups 1,” with the final search completed in May 2013. Studies from the references of the related reports were checked. Articles in all languages were searched to ensure the relevant studies were not missed. The following inclusion criteria were applied: (1) case-control studies or nested case-control studies within cohort studies, if any (2) studies evaluating association between XRCC1 polymorphism and leukemia risk, (3) full text reports which including enough data to calculate odds ratios (ORs) and 95% confidence intervals (CIs). The exclusion criteria were as follows: (1) duplicated reports, (2) reviews or meta-analyses, if they were performed without additional eligible studies; otherwise, the additional eligible study was included in our meta-analysis, (3) if the same population was used in multiple studies, only the most complete or latest study was selected for further analysis.

### Data extraction procedure

According to the study identification criteria, the available studies were reviewed, selected, and the following information from the eligible studies was extracted: first author’s last name, publication year, country, leukemia type, number of case and control subjects, ethnicity, control subjects source population, and genotype numbers of cases and controls.

### Study quality assessment

Critical quality assessment of the included studies was performed by Effective Public Health Practice Project Quality Assessment Tool (EPHPP). With this tool, assessments of the risk of bias or methodological quality were made separately for six individual domains: selection bias, study design, confounders, blinding, data collection method, and withdrawals and drop-outs. The comprehensive dictionary for the assessment tool was used to guide the rating of the studies. Each domain was rated as strong, moderate, or weak. The study quality was then evaluated as strong, moderate, or weak if there were no, one, or two or more in total weak ratings for all the domains, respectively [6]. Two reviewers (Haijun Zhang and Hang Liu) independently reviewed the studies and they resolved discrepancies through discussion.

### Statistical analysis

Statistical analyses were performed as described previously [7], [8]. Briefly, for individual studies, Hardy-Weinberg equilibrium of control subjects was tested by Pearson’s goodness-of-fit *χ* ^2^ test. The strength of association between XRCC1 Arg399Gln, Arg194Trp, and Arg280His polymorphisms and leukemia risk was measured by odds ratios (ORs) and the corresponding 95% confidence intervals (CIs). We used codominant, dominant, and recessive genetic models to assess the pooled ORs. For both Arg399Gln and Arg280His (G>A) polymorphisms, the codominant model included homozygous comparison of AA vs. GG and heterozygous comparison of GA vs. GG, the dominant model was AA+GA vs. GG, and the recessive model was AA vs. GA+GG. For Arg194Trp (C>T) polymorphism, the codominant model included homozygous comparison of TT vs. CC and heterozygous comparison of CT vs. CC, the dominant model was TT+CT vs. CC, and the recessive model was TT vs. CT+CC. Stratified analyses were performed by race, control source, and tumor type. Due to the high heterogeneity across the studies, the random effects model based on the DerSimonian and Laird method was applied [9]. For calculating the OR of a subgroup containing a single stud, the inverse variance method was used instead. Publication bias was evaluated by Begg’s rank correlation test [10] and Egger’s linear regression test [11]. If the publication bias tests indicated bias existed, the Duval and Tweedie “trim and fill” method was used to adjust the bias [12]. The statistical power for XRCC1 polymorphisms and leukemia risk in three genetic models was calculated by OpenEpi program (Version 2.3.1, www.OpenEpi.com). Statistical analysis was conducted in R 2.15.2 using “meta” package [13].

## Results

### Study identification and characteristics of studies

**Figure 1:**
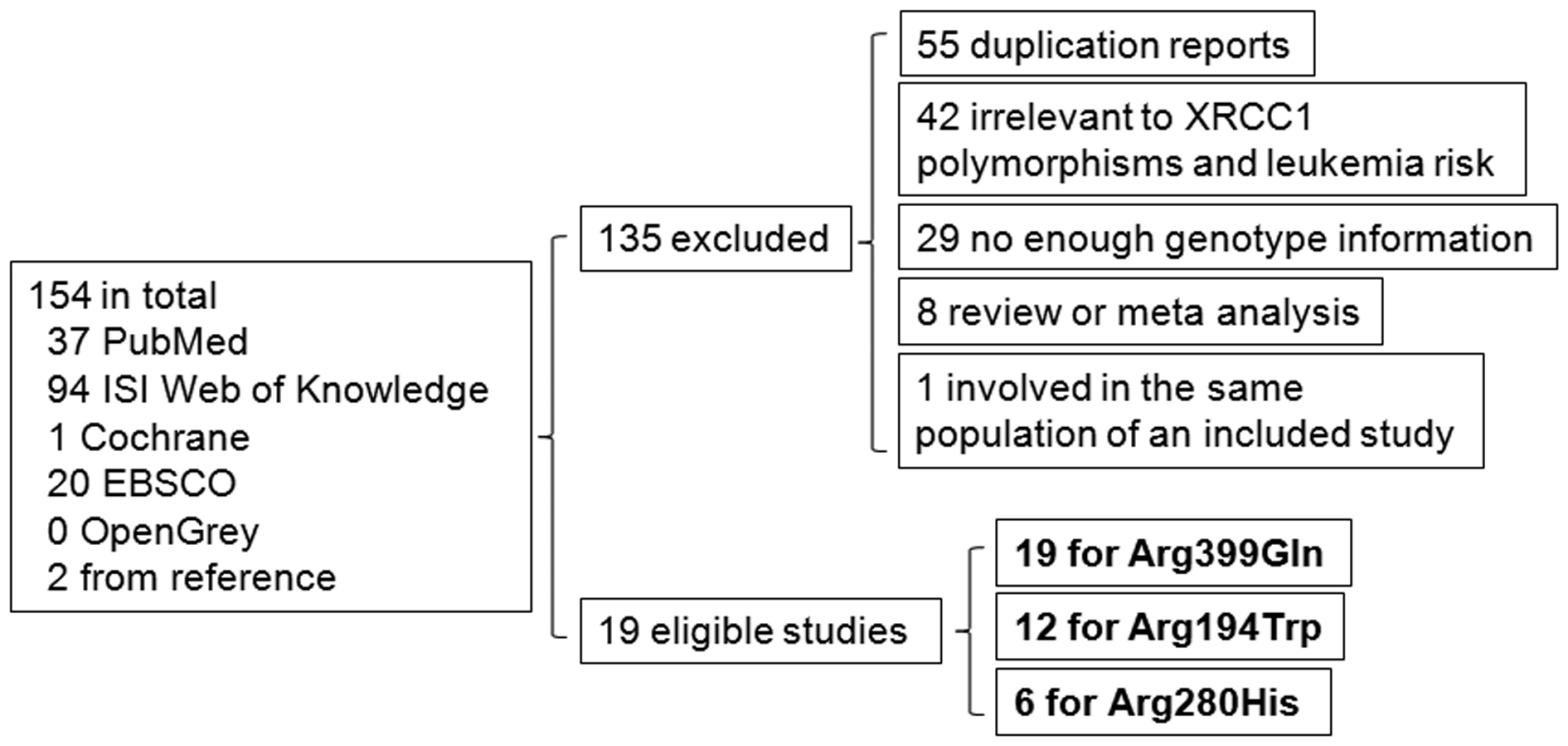
Flow diagram of study identification process.

Searches of PubMed, ISI Web of Knowledge, Cochrane, EBSCO, and OpenGrey databases and manually searching references returned 154 studies. Among them, 135 reports were excluded for the following reasons: 55 were duplications, 42 were irrelevant to XRCC1 polymorphisms and leukemia risk, 29 did not provide enough genotype information, and 8 were reviews or meta-analyses., In addition, studies that involve the same population as another eligible study were excluded to avoid bias [14]. Also of note was Annamaneni et al. report [15], in which the minor allele frequencies of Arg194Trp polymorphism in both case and control groups were much higher than major allele frequencies, which indicated the genotype numbers of the Arg194Trp polymorphism may include errors in this study. We therefore excluded this study from Arg194Trp analysis. After removing these ineligible studies, a total 19 studies with 3387 cases and 6168 controls for Arg399Gln (G>A) polymorphism [15], [16], [17], [18], [19], [20], [21], [22], [23], [24], [25], [26], [27], [28], [29], [30], [31], [32], [33], 12 studies with 2043 cases and 4550 controls for Arg194Trp (C>T) [17], [18], [20], [21], [22], [23], [24], [25], [26], [28], [29], [32], and 6 studies with 1445 cases and 1905 controls for Arg280His (G>A) [15], [22], [23], [26], [28], [30] were included for further analysis (Figure 1). A database was established to display the study characteristics of each eligible study (Table 1). The distribution of genotypes in the control group of each study was in agreement with Hardy-Weinberg equilibrium except for 4 studies for Arg399Gln, 3 studies for Arg194Trp and 1 study for Arg280His (Table 1). All the included studies were rated as strong or moderate by EPHPP Quality Assessment Tool, indicating that the synthesis of results in a meta-analysis should be reliable (Table 1).

**Table 1:** Study characteristics of the meta-analysis.

Polymorphism	Author	Year	Country	Racial descent	Tumor type(# of cases)	Control source	Case			Control				HWE	QA
Arg399Gln							GG	GA	AA		GG	GA	AA		
(G>A)	Seedhouse	2002	UK	Caucasian	AML (167)	Population	70	69	28		55	76	47	0.05	Strong
	Joseph	2005	India	Asian	ALL (117)	Hospital	55	46	16		75	33	9	0.06	Strong
	Matullo	2006	Multiple	Unknown	Unknown (169)	Population	67	74	28		484	482	128	0.63	Moderate
	Deligezer	2007	Turkey	Caucasian	AML (72), CML (182)	Population	103	121	30		96	101	29	0.76	Moderate
	Pakakasama	2007	Thailand	Asian	ALL (108)	Population	39	60	9		175	124	18	0.51	Strong
	Batar	2009	Turkey	Caucasian	ALL (70)	Population	24	37	9		24	37	14	0.97	Strong
	Ganster	2009	Austria	Unknown	CLL (429)	Population	173	192	64		184	193	52	0.90	Moderate
	Meza-Espinoza	2009	Mexico	Unknown	ALL (120)	Population	57	51	12		65	47	8	0.90	Moderate
	Tumer	2010	Turkey	Caucasian	ALL (167)	Population	63	77	27		92	78	20	0.57	Strong
	Shi	2011	China	Asian	AML (306)	Population	173	114	19		316	213	29	0.37	Strong
	Stanczyk	2011	Poland	Caucasian	ALL (97)	Population	34	45	18		50	57	24	0.28	Moderate
	Canalle	2011	Brazil	Caucasian,unknown	ALL (201)	Hospital	112	72	17		186	152	23	0.27	Strong
	Özcan	2011	Turkey	Caucasian	AML (36), ALL (9)	Population	22	22	1		42	43	15	0.47	Strong
	Duman	2012	Turkey	Caucasian	CLL (73)	Population	7	50	16		19	26	5	0.36	Moderate
	Kim	2012	Korea	Asian	AML (415)	Population	234	155	26		914	693	91	0.01	Strong
	Abramenko	2012	Ukraine	Caucasian	CLL (169)	Population	67	82	20		38	41	15	0.48	Strong
	El-Din	2012	Egypt	Caucasian	AML (40)	Population	20	16	4		16	2	2	0.01	Strong
	Annamaneni	2012	India	Asian	CML (350)	Population	79	191	80		61	235	54	<0.01	Strong
	Sorour	2013	Egypt	Caucasian	AML (90)	Population	54	27	9		33	27	0	0.02	Strong
Arg194Trp							CC	CT	TT		CC	CT	TT		
(C>T)	Seedhouse	2002	UK	Caucasian	AML (126)	Population	112	14	0		78	7	2	<0.01	Strong
	Joseph	2005	India	Asian	ALL (117)	Hospital	77	32	8		91	22	4	0.09	Strong
	Matullo	2006	Multiple	Unknown	Unknown (169)	Population	145	23	1		951	141	2	0.17	Moderate
	Pakakasama	2007	Thailand	Asian	ALL (108)	Population	62	44	2		150	145	22	0.10	Strong
	Batar	2009	Turkey	Caucasian	ALL (70)	Population	52	16	2		64	11	0	0.49	Strong
	Ganster	2009	Austria	Unknown	CLL (439)	Population	371	63	5		389	45	5	0.01	Moderate
	Meza-Espinoza	2009	Mexico	Unknown	ALL (120)	Population	80	34	6		86	31	3	0.92	Moderate
	Tumer	2010	Turkey	Caucasian	ALL (167)	Population	140	27	0		159	26	5	0.01	Strong
	Canalle	2011	Brazil	Caucasian,unknown	ALL (201)	Hospital	168	32	1		298	59	4	0.58	Strong
	Duman	2012	Turkey	Caucasian	CLL (73)	Population	64	8	1		41	9	0	0.48	Moderate
	Kim	2012	Korea	Asian	AML (413)	Population	167	208	38		775	741	164	0.50	Strong
	El-Din	2012	Egypt	Caucasian	AML (40)	Population	11	14	15		14	4	2	0.09	Strong
Arg280His							GG	GA	AA		GG	GA	AA		
(G>A)	Joseph	2005	India	Asian	ALL (117)	Hospital	76	38	3		85	30	2	0.73	Strong
	Pakakasama	2007	Thailand	Asian	ALL (108)	Population	94	14	0		272	42	3	0.34	Strong
	Ganster	2009	Austria	Unknown	CLL (443)	Population	396	47	0		388	53	2	0.90	Moderate
	Meza-Espinoza	2009	Mexico	Unknown	ALL (120)	Population	87	31	2		88	31	1	0.33	Moderate
	Shi	2011	China	Asian	AML (307)	Population	236	66	5		445	109	4	0.34	Strong
	Annamaneni	2012	India	Asian	CML (350)	Population	346	4	0		338	11	1	0.01	Strong

AML, acute myeloid leukemia; ALL, acute lymphocytic leukemia; CML, chronic myeloid leukaemia; CLL, chronic lymphocytic leukemia; HWE, *P* value of Pearson’s goodness-of-fit χ2 test for Hardy-Weinberg equilibrium; QA, quality assessment; Unknown, including study populations in which the race was mixed/unclear or tumor type was not described.

### Meta-analysis results

Meta-analysis and relevant subgroups analysis by tumor type, race and control sources were conducted to examine the association between XRCC1 Arg399Gln (G>A), Arg194Trp (C>T), and Arg280His (G>A) polymorphisms and leukemia risk in three genetic models. Stratified analysis by race and control sources in each leukemia type and by etiology in AML was further performed to explore the possible associations. The analyses results were shown in Table 2. The statistical power for XRCC1 polymorphisms and leukemia risk in three genetic models was shown in Table 3.

**Table 2:** Pooled ORs and 95% CIs for XRCC1 Arg399Gln, Arg194Trp and Arg280His meta-analysis.

Polymorphism	Study group	n	Codominant				Dominant		Recessive	
Arg399Gln			AA vs. GG		GA vs. GG		AA + GA vs. GG		AA vs. GA + GG	
(G>A)			OR (95% CI)	*P*	OR (95% CI)	*P*	OR (95% CI)	*P*	OR (95% CI)	*P*
	Total	19	1.25 (0.98–1.59)	0.08	1.10 (0.93–1.31)	0.27	1.13 (0.96–1.34)	0.15	1.19 (0.98–1.45)	0.08
	Racial decent									
	Asian	5	1.31 (1.00–1.71)	0.05	1.12 (0.77–1.64)	0.55	1.18 (0.83–1.69)	0.35	1.42 (1.12–1.81)	<0.01
	Caucasian	11	1.10 (0.68–1.78)	0.69	1.12 (0.86–1.47)	0.40	1.12 (0.85–1.48)	0.44	0.97 (0.68–1.38)	0.87
	Unknown	4	1.44 (1.07–1.93)	0.02	1.04 (0.80–1.35)	0.77	1.12 (0.90–1.39)	0.32	1.39 (1.06–1.84)	0.02
	Control source									
	Population	17	1.21 (0.93–1.57)	0.16	1.10 (0.92–1.31)	0.32	1.12 (0.94–1.33)	0.21	1.16 (0.94–1.43)	0.18
	Hospital	2	1.62 (0.84–3.11)	0.15	1.19 (0.50–2.82)	0.69	1.27 (0.54–2.98)	0.58	1.54 (0.91–2.58)	0.11
	Tumor type									
	AML	7	0.94 (0.56–1.56)	0.80	0.92 (0.73–1.15)	0.46	0.92 (0.73–1.16)	0.46	0.94 (0.60–1.49)	0.80
	Etiology									
	*De novo*	4	1.12 (0.48–2.59)	0.79	0.96 (0.59–1.56)	0.87	1.00 (0.65–1.54)	0.99	1.07 (0.50–2.29)	0.86
	Secondary	2	0.30 (0.11–0.82)	0.02	0.60 (0.35–1.03)	0.06	0.53 (0.31–0.89)	0.02	0.40 (0.15–1.06)	0.07
	Racial decent									
	Asian	2	1.14 (0.79–1.65)	0.47	0.91 (0.76–1.09)	0.31	0.94 (0.79–1.11)	0.47	1.19 (0.83–1.70)	0.34
	Caucasian	5	0.83 (0.32–2.16)	0.71	0.97 (0.60–1.57)	0.90	0.94 (0.59–1.51)	0.80	0.79 (0.35–1.76)	0.56
	Control source									
	Population	7	0.94 (0.56–1.56)	0.80	0.92 (0.73–1.15)	0.46	0.92 (0.73–1.16)	0.46	0.94 (0.60–1.49)	0.80
	ALL	8	1.50 (1.11–2.02)	0.01	1.32 (0.99–1.75)	0.06	1.35 (1.02–1.78)	0.03	1.31 (0.99–1.74)	0.06
	Racial decent									
	Asian	2	2.33 (1.25–4.34)	0.01	2.06 (1.44–2.95)	< 0.01	2.11 (1.50–2.97)	< 0.01	1.69 (0.93–3.07)	0.09
	Caucasian	5	1.27 (0.86–1.86)	0.23	1.10 (0.86–1.41)	0.45	1.13 (0.89–1.43)	0.31	1.16 (0.81–1.66)	0.41
	Unknown	2	1.55 (0.70–3.43)	0.28	0.76 (0.25–2.28)	0.62	0.88 (0.36–2.16)	0.78	1.61 (0.74–3.47)	0.23
	Control source									
	Population	6	1.47 (1.02–2.11)	0.04	1.45 (1.15–1.83)	<0.01	1.45 (1.13–1.85)	<0.01	1.23 (0.88–1.72)	0.23
	Hospital	2	1.62 (0.84–3.11)	0.15	1.19 (0.50–2.82)	0.69	1.27 (0.54–2.98)	0.58	1.54 (0.91–2.58)	0.11
	CML	2	1.05 (0.72–1.55)	0.78	0.81 (0.48–1.36)	0.43	0.86 (0.61–1.21)	0.38	1.26 (0.70–2.26)	0.45
	Racial decent									
	Asian	1	1.14 (0.71–1.85)	0.58	0.63 (0.43–0.92)	0.02	0.72 (0.50–1.05)	0.09	1.62 (1.11–2.38)	0.01
	Caucasian	1	0.91 (0.48–1.73)	0.78	1.06 (0.70–1.61)	0.77	1.03 (0.69–1.53)	0.88	0.89 (0.49–1.61)	0.69
	Control source									
	Population	2	1.05 (0.72–1.55)	0.78	0.81 (0.48–1.36)	0.43	0.86 (0.61–1.21)	0.38	1.26 (0.70–2.26)	0.45
	CLL	3	1.73 (0.64–4.67)	0.28	1.58 (0.79–3.14)	0.20	1.61 (0.79–3.27)	0.19	1.21 (0.69–2.10)	0.51
	Racial decent									
	Caucasian	2	2.41 (0.22–26.39)	0.47	2.30 (0.52–10.22)	0.27	2.32 (0.43–12.54)	0.33	1.25 (0.36–4.35)	0.72
	Unknown	1	1.31 (0.86–1.99)	0.21	1.06 (0.79–1.41)	0.70	1.11 (0.85–1.46)	0.45	1.27 (0.86–1.88)	0.23
	Control source									
	Population	3	1.73 (0.64–4.67)	0.28	1.58 (0.79–3.14)	0.20	1.61 (0.79–3.27)	0.19	1.21 (0.69–2.10)	0.51
Arg194Trp			TT vs. CC		CT vs. CC		TT + CT vs. CC		TT vs. CT + CC	
(C>T)			OR (95% CI)	*P*	OR (95% CI)	*P*	OR (95% CI)	*P*	OR (95% CI)	*P*
	Total	12	1.21 (0.65–2.27)	0.55	1.20 (1.00–1.43)	0.05	1.20 (0.96–1.48)	0.10	1.11 (0.63–1.94)	0.72
	Racial decent									
	Asian	3	0.92 (0.33–2.53)	0.87	1.16 (0.75–1.78)	0.50	1.13 (0.69–1.85)	0.62	0.87 (0.36–2.08)	0.75
	Caucasian	6	1.06 (0.20–5.68)	0.94	1.22 (0.81–1.83)	0.34	1.27 (0.77–2.08)	0.35	0.97 (0.22–4.31)	0.97
	Unknown	4	1.61 (0.69–3.74)	0.27	1.28 (0.98–1.66)	0.07	1.29 (1.00–1.67)	0.05	1.54 (0.66–3.57)	0.31
	Control source									
	Population	10	1.18 (0.56–2.47)	0.66	1.19 (0.97–1.47)	0.09	1.19 (0.93–1.52)	0.17	1.08 (0.56–2.08)	0.82
	Hospital	2	1.32 (0.28–6.33)	0.73	1.24 (0.70–2.18)	0.46	1.27 (0.66–2.45)	0.48	1.27 (0.31–5.19)	0.74
	Tumor type									
	AML	3	1.48 (0.24–9.08)	0.67	1.53 (0.92–2.55)	0.10	1.74 (0.79–3.86)	0.17	1.21 (0.27–5.42)	0.80
	Etiology									
	*De novo*	2	1.49 (0.03–87.31)	0.85	2.21 (0.69–7.09)	0.18	2.43 (0.43–13.83)	0.32	1.16 (0.04–37.87)	0.94
	Secondary	1	1.26 (0.06–27.73)	0.89	1.86 (0.34–10.01)	0.47	1.44 (0.28–7.51)	0.66	1.18 (0.05–25.84)	0.92
	Racial decent									
	Asian	1	1.08 (0.73–1.59)	0.72	1.30 (1.04–1.63)	0.02	1.26 (1.01–1.57)	0.04	0.94 (0.65–1.36)	0.73
	Caucasian	2	1.40 (0.02–92.08)	0.87	2.24 (0.73–6.86)	0.16	2.47 (0.45–13.49)	0.30	1.08 (0.03–39.94)	0.97
	Control source									
	Population	3	1.48 (0.24–9.08)	0.67	1.35 (1.09–1.68)	0.10	1.74 (0.79–3.86)	0.17	1.21 (0.27–5.42)	0.80
	ALL	6	0.88 (0.28–2.77)	0.82	1.11 (0.85–1.45)	0.46	1.12 (0.80–1.55)	0.51	0.87 (0.30–2.52)	0.79
	Racial decent									
	Asian	2	0.75 (0.07–7.97)	0.81	1.09 (0.48–2.51)	0.84	1.08 (0.40–2.89)	0.88	0.75 (0.09–6.12)	0.79
	Caucasian	3	0.60 (0.07–5.11)	0.64	1.14 (0.79–1.64)	0.48	1.10 (0.71–1.68)	0.68	0.58 (0.07–4.68)	0.61
	Unknown	2	2.08 (0.57–7.63)	0.27	1.27 (0.78–2.08)	0.34	1.32 (0.82–2.13)	0.25	1.97 (0.54–7.18)	0.30
	Control source									
	Population	4	0.71 (0.13–3.97)	0.70	1.06 (0.75–1.50)	0.75	1.05 (0.69–1.61)	0.82	0.72 (0.14–3.64)	0.69
	Hospital	2	1.32 (0.28–6.33)	0.73	1.24 (0.70–2.18)	0.46	1.27 (0.66–2.45)	0.48	1.27 (0.31–5.19)	0.74
	CLL	2	1.14 (0.35–3.63)	0.83	1.03 (0.42–2.53)	0.94	1.10 (0.53–2.29)	0.80	1.10 (0.34–3.52)	0.87
	Racial decent									
	Caucasian	1	1.93 (0.08–48.52)	0.69	0.57 (0.20–1.59)	0.28	0.64 (0.23–1.75)	0.38	2.09 (0.08–52.34)	0.65
	Unknown	1	1.05 (0.30–3.65)	0.94	1.47 (0.98–2.21)	0.07	1.43 (0.96–2.11)	0.08	1.00 (0.29–3.48)	1.00
	Control source									
	Population	2	1.14 (0.35–3.63)	0.83	1.03 (0.42–2.53)	0.94	1.10 (0.53–2.29)	0.80	1.10 (0.34–3.52)	0.87
Arg280His			AA vs. GG		GA vs. GG		AA + GA vs GG		AA vs GA + GG	
(G>A)			OR (95% CI)	*P*	OR (95% CI)	*P*	OR (95% CI)	*P*	OR (95% CI)	*P*
	Total	6	1.32 (0.56–3.11)	0.52	1.02 (0.82–1.27)	0.87	1.01 (0.78–1.30)	0.97	1.28 (0.55–3.01)	0.57
	Racial decent									
	Asian	4	1.50 (0.57–3.91)	0.41	1.05 (0.73–1.51)	0.79	1.02 (0.68–1.54)	0.91	1.44 (0.55–3.75)	0.46
	Unknown	2	0.76 (0.08–7.39)	0.81	0.92 (0.65–1.28)	0.61	0.90 (0.65–1.26)	0.55	0.77 (0.08–7.31)	0.82
	Control source									
	Population	5	1.23 (0.47–3.26)	0.67	0.97 (0.78–1.22)	0.81	0.94 (0.72–1.24)	0.68	1.22 (0.46–3.22)	0.69
	Hospital	1	1.68 (0.27–10.31)	0.58	1.42 (0.80–2.50)	0.23	1.43 (0.82–2.50)	0.21	1.51 (0.25–9.23)	0.65
	Tumor type									
	AML	1	2.36 (0.63–8.86)	0.20	1.14 (0.81–1.61)	0.45	1.18 (0.85–1.66)	0.32	2.29 (0.61–8.60)	0.22
	Etiology									
	*De novo*	1	1.58 (0.35–7.13)	0.55	1.10 (0.77–1.58)	0.59	1.12 (0.79–1.59)	0.53	1.55 (0.34–6.98)	0.57
	Secondary	1	8.90 (1.56–50.94)	0.01	1.47 (0.67–3.24)	0.34	1.73 (0.83–3.63)	0.14	8.15 (1.44–46.06)	0.02
	Racial decent									
	Asian	1	2.36 (0.63–8.86)	0.20	1.14 (0.81–1.61)	0.45	1.185 (0.85–1.66)	0.32	2.29 (0.61–8.60)	0.22
	Control source									
	Population	1	2.36 (0.63–8.86)	0.20	1.14 (0.81–1.61)	0.45	1.185 (0.85–1.66)	0.32	2.29 (0.61–8.60)	0.22
	ALL	3	1.35 (0.37–4.98)	0.65	1.13 (0.80–1.59)	0.49	1.13 (0.80–1.58)	0.49	1.28 (0.35–4.71)	0.71
	Racial decent									
	Asian	2	1.15 (0.24–5.39)	0.86	1.20 (0.78–1.84)	0.41	1.17 (0.74–1.84)	0.50	1.07 (0.23–5.00)	0.94
	Unknown	1	2.02 (0.18–22.72)	0.57	1.01 (0.57–1.81)	0.97	1.04 (0.59–1.84)	0.88	2.02 (0.18–22.54)	0.57
	Control source									
	Population	2	1.07 (0.16–7.01)	0.94	0.99 (0.64–1.53)	0.97	0.98 (0.64–1.50)	0.92	1.07 (0.17–7.00)	0.94
	Hospital	1	1.68 (0.27–10.31)	0.58	1.42 (0.80–2.51)	0.23	1.43 (0.82–2.50)	0.21	1.51 (0.25–9.23)	0.65
	CML	1	0.33 (0.01–8.02)	0.49	0.36 (0.11–1.13)	0.08	0.33 (0.10–1.02)	0.05	0.33 (0.01–8.19)	0.50
	Racial decent									
	Asian	1	0.33 (0.01–8.02)	0.49	0.36 (0.11–1.13)	0.08	0.33 (0.10–1.02)	0.05	0.33 (0.01–8.19)	0.50
	Control source									
	Population	1	0.33 (0.01–8.02)	0.49	0.36 (0.11–1.13)	0.08	0.33 (0.10–1.02)	0.05	0.33 (0.01–8.19)	0.50
	CLL	1	0.20 (0.01–4.10)	0.29	0.87 (0.57–1.32)	0.51	0.84 (0.55–1.27)	0.40	0.20 (0.01–4.16)	0.30
	Racial decent									
	Unknown	1	0.20 (0.01–4.10)	0.29	0.87 (0.57–1.32)	0.51	0.84 (0.55–1.27)	0.40	0.20 (0.01–4.16)	0.30
	Control source									
	Population	1	0.20 (0.01–4.10)	0.29	0.87 (0.57–1.32)	0.51	0.84 (0.55–1.27)	0.40	0.20 (0.01–4.16)	0.30

n, number of studies; unknown, including study populations in which the race was mixed or unclear; secondary, including secondary and therapy-related AML; OR, odds ratio; CI, confidence interval; *P*, *P* value.

**Table 3:** Statistical power (%) for XRCC1 polymorphisms and leukemia risk.

Polymorphism		Homozygous codominant	Heterozygous codominant	Dominant	Recessive
Arg399Gln (G>A)		AA vs. GG	GA vs. GG	AA+GA vs. GG	AA vs. GA+GG
		100	80.4	98.8	99.8
Arg194Trp (C>T)		TT vs. CC	TC vs. CC	TT+TC vs. CC	TT vs. TC+CC
		36.7	50.4	65.4	30.1
Arg280His (G>A)		AA vs. GG	GA vs. GG	AA+GA vs. GG	AA vs. GA+GG
		1.1	8.0	7.9	1.1

### XRCC1 Arg399Gln (G>A) polymorphism

In the overall analysis, no statistically significant association between XRCC1 Arg399Gln polymorphism and leukemia susceptibility was observed in three genetic models. A very mild publication bias was detected by Egger’s test (*P*  =  0.04; Begg’s test, *P*  =  0.12) in the codominant heterozygous comparison (GA vs. GG) (other data not shown). We repeated meta-analysis with the “trim and fill” method to adjust publication bias in this genetic model. The conclusion was not influenced, which indicated the robustness our conclusions (data not shown). In the stratified analysis by racial descent, increased risk of leukemia was found among Asians (AA vs. GA+GG, OR  =  1.42, 95% CI: 1.12-1.81), and the unknown or mixed race subgroup (AA vs. GG, OR  =  1.44, 95% CI: 1.07-1.93; AA vs. GA+GG, OR  =  1.39, 95% CI: 1.06-1.84).

**Figure 2:**
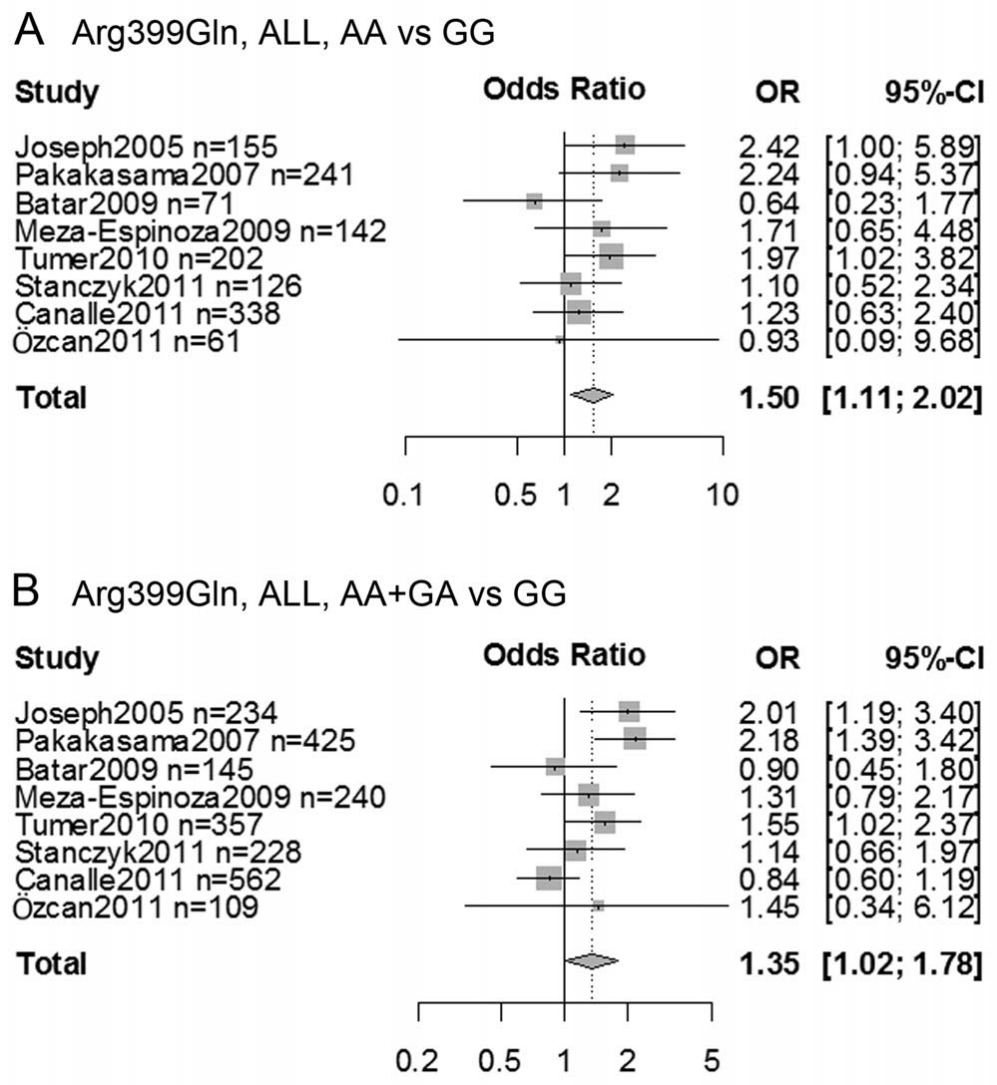
Forest plots showed meta-analysis of XRCC1 Arg399Gln polymorphism and acute lymphocytic leukemia (ALL) risk in (A) homozygous codominant (AA vs. GG), and (B) dominant (AA+GA vs. GG) models. OR, Odds ratio; CI, confidence interval.

When performing meta-analysis by tumor type, higher risk can be detect in ALL (AA vs. GG, OR  =  1.50, 95% CI: 1.11-2.02; AA+GA vs. GG, OR  =  1.35, 95% CI: 1.02-1.78) (Figure 2A and 2B) and among ALL Asian (AA vs. GG, OR  =  2.33, 95% CI: 1.25-4.34; GA vs. GG, OR  =  2.06, 95% CI: 1.44-2.95; AA+GA vs. GG, OR  =  2.11, 95% CI: 1.50-2.97) and population-based control subgroups (AA vs. GG, OR  =  1.47, 95% CI: 1.02-2.11; GA vs. GG, OR  =  1.45, 95% CI: 1.15-1.83; AA+GA vs. GG, OR  =  1.45, 95% CI: 1.13-1.85). Although significant association was not found in AML, CML and CLL and in most subgroups by race and control sources of these leukemia types, a protective effect was exhibited among the secondary and therapy-related AML (AA vs. GG, OR  =  0.30, 95% CI: 0.11-0.82; AA+GA vs. GG, OR  =  0.53, 95% CI: 0.31-0.89) and the CML Asian subjects (GA vs. GG, OR  =  0.63, 95% CI: 0.43-0.92; AA vs. GA+GG, OR  =  1.62, 95% CI: 1.11-2.38).

### XRCC1 Arg194Trp (C>T) polymorphism

XRCC1 Arg194Trp was not associated with the leukemia susceptibility in the overall population or in different race and control source subgroups. Publication bias does not exist across the studies (data not shown). Arg194Trp worked as a risk factor in the AML Asians (CT vs. CC, OR  =  1.30, 95% CI: 1.04-1.63; TT+CT vs. CC, OR  =  1.26, 95% CI: 1.01-1.57) albeit there was only one study for this race subgroup.

### XRCC1 Arg280His (G>A) polymorphism

As for Arg280His polymorphism, no statistically significant association was present in any genetic model or subgroup except for among the secondary and therapy-related AML codominant model homozygous comparison (AA vs. GG, OR  =  8.90, 95% CI: 1.56-50.94), and the recessive model (AA vs. GA+GG, OR  =  8.15, 95% CI: 1.44-46.06). Begg’s test and Egger’s test showed publication bias in the main meta-analysis in the codominant homozygous comparison (AA vs. GG, Begg’s test *P*  =  0.04, Egger’s test *P*  =  0.02), and the recessive model (AA vs. GA+GG, Begg’s test *P*  =  0.04, Egger’s test *P*  =  0.02) (other data not shown). Adjusting these two models by “trim and fill” method did not influence the conclusion (data not shown).

## Discussion

Nonsynonymous XRCC1 polymorphisms Arg399Gln, Arg194Trp, and Arg280His have been implicated in the risk of various cancers [7], [8], [34]. The relationship between these XRCC1 polymorphisms and leukemia risk has been examined in some case­-control studies, but the results of these studies were contradictory and inconclusive. Although the association between XRCC1 polymorphisms and risk of some types of leukemia was recognized by a number of studies [15], [17], [20], [21], [22], [23], [24], [25], [27], [28], [29], [32], [33], other reports did not take the XRCC1 genetic variants as risk or protective factors for leukemia [16], [18], [19], [26], [30], [31]. We conducted a meta-analysis that includes 19 studies for Arg399Gln (G>A) polymorphism, 12 studies for Arg194Trp (C>T), and 6 studies for Arg280His (G>A) to evaluate XRCC1 genotype-leukemia association (Figure 1).

Although associations between Arg399Gln, Arg194Trp, and Arg280His and overall leukemia risks were lacking, higher leukemia susceptibility was detected for Arg399Gln among Asians (AA vs. GA+GG, OR  =  1.42, 95% CI: 1.12-1.81), and the unknown or mixed race subgroup (AA vs. GG, OR  =  1.44, 95% CI: 1.07-1.93; AA vs. GA+GG, OR  =  1.39, 95% CI: 1.06-1.84) while no such effect was found among Caucasians (Table 2). The finding in Asians is consistent with previous studies that the Arg399Gln polymorphism increases glioma risk among Asians [8] but does not alter glioma [8] or skin cancer [7] risks among Caucasians, which indicates a race-specific effect of this polymorphism in some tumors. The analysis for unknown or mixed population implies that, excepting Caucasians, other races in these populations may be sensitive to Arg399Gln associated leukemia risk. Collecting more samples from races other than Asian and Caucasian will be necessary to verify the conclusion in future studies.

The predisposition of each leukemia type could be differentially influenced by genetic factors. We performed stratified analysis by leukemia type in order to clarify the role of XRCC1 polymorphisms in the development of individual types of leukemia (Table 2). In the secondary and therapy-related AML, Arg399Gln is a protective factor (AA vs. GG, OR  =  0.30, 95% CI: 0.11-0.82; AA+GA vs. GG, OR  =  0.53, 95% CI: 0.31-0.89) whereas Arg280His is a risk factor (AA vs. GG, OR  =  8.90, 95% CI: 1.56-50.94; AA vs. GA+GG, OR  =  8.15, 95% CI: 1.44-46.06). The occurrence of secondary and therapy-related AML is correlated with prior chemotherapy and/or radiation therapy, which probably involves XRCC1 mediated DNA repair. XRCC1 polymorphisms could alter the susceptibility of this AML category by changing XRCC1 DNA repair capacity. However, only one or two studies were included in these subgroups, which compromises the reliability of these findings. Arg399Gln increases CML risk (GA vs. GG, OR  =  0.63, 95% CI: 0.43-0.92; AA vs. GA+GG, OR  =  1.62, 95% CI: 1.11-2.38), and Arg194Trp increases AML risk (CT vs. CC, OR  =  1.30, 95% CI: 1.04-1.63; TT+CT vs. CC, OR  =  1.26, 95% CI: 1.01-1.57) among Asians. The conclusions are both derived from one single study and need to be interpreted carefully. Arg399Gln is associated with higher risk in ALL (AA vs. GG, OR  =  1.50, 95% CI: 1.11-2.02; AA+GA vs. GG, OR  =  1.35, 95% CI: 1.02-1.78) (Figure 2A and 2B) and among ALL Asian (AA vs. GG, OR  =  2.33, 95% CI: 1.25-4.34; GA vs. GG, OR  =  2.06, 95% CI: 1.44-2.95; AA+GA vs. GG, OR  =  2.11, 95% CI: 1.50-2.97) and ALL population-based control subgroups (AA vs. GG, OR  =  1.47, 95% CI: 1.02-2.11; GA vs. GG, OR  =  1.45, 95% CI: 1.15-1.83; AA+GA vs. GG, OR  =  1.45, 95% CI: 1.13-1.85). The results from population-based and hospital-based studies are different. The former may be more reliable since patient controls usually carry other disease conditions, which might potentially influence leukemia risk.

The advantages of this meta-analysis are that it is the most complete and the information from the eligible studies is utilized as much as possible through genetic model and stratified analysis. However, there are several limitations in this study. First, expression of specific genes is highly regulated by a transcriptional control mechanism requiring transcription factors-gene promoter interaction [35], [36], [37], [38]. XRCC1 -77 T>C polymorphism might change the binding capacity of the transcription factor SP1 to the XRCC1 promoter, and then downregulate XRCC1 expression [39]. This polymorphism contributes to the development of lung cancer [40] and breast cancer [39]. However, studies addressing the association between XRCC1 -77 T>C polymorphism and leukemia risk are absent, and cannot be analyzed by our meta-analysis. In addition to the promoter control, the regulation of 3’ untranslated region (3’UTR), by regulators for example microRNAs, influences gene expression in cancer and developmental process [41], [42]. However, whether the XRCC1 polymorphisms in 3’UTR alter leukemia risk was not fully studied. Second, although the statistical power for Arg399Gln was greater than 80% in all three genetic models, the power for Arg194Trp and Arg280His was not as high as that in Arg399Gln (Table 3). The lower power for these two polymorphisms indicates that the weak associations between Arg194Trp and Arg280His and leukemia risk might not be detected while the associations that are detected in our meta-analysis still remain interesting. Statistical power in the genetic association study is primarily affected by the participant numbers and the effect size (OR) when choosing conventional level of *α* significance criterion 0.05 in the target population. Because the effects of the Arg194Trp and Arg280His polymorphisms on leukemia risk are weak (Table 2, ORs are close to 1), further study with larger sample numbers will be helpful to improve the power for detecting the positive effects. To the current meta-analysis, the negative association between these two polymorphisms and leukemia susceptibility should be cautiously interpreted. Third, the eligible study number for some individual subgroups is small, which restricts the application of the conclusions drawn from those subgroups. Fourth, four eligible studies contained an "unknown" race population. Specific race information was not collect by two of them [22], [25]. The other two studies included non-Caucasian mestizos or mulattos [18], [26]. Since the ethnicities of mestizos and mulattos were difficult to determine, we then described the non-Caucasian population in these two studies as "unknown" race. We have discussed the necessity of performing study with different races in the future studies according to the findings from the unknown population. In the report of Matullo et al. [25], information about the leukemia subtype was not presented. The purpose of the original study was not to conduct a detailed analysis of the subtypes of leukemia. In addition to leukemia, multiple types of cancer as well as emphysema and chronic obstructive pulmonary disease were evaluated based on the data collected by 23 centers from 10 countries. It would be difficult for the researchers to extract the detailed subtype information for a single cancer type, for example leukemia, after the study have been completed. Moreover, the case numbers of this study were not very large; thus the study would not strongly affect the outcome of our subgroup analysis by tumor type of leukemia. Accordingly, we did not use the leukemia tumor type information of this study in our meta-analysis. Finally, African populations have not been well studied previously regarding XRCC1 polymorphisms and leukemia susceptibility. Data from this population will be useful to establish a better overview of polymorphism-leukemia association.

Taken together, results from this meta-analysis demonstrate that XRCC1 Arg399Gln, Arg194Trp and Arg280His polymorphisms might not be associated with overall leukemia risk. However, these polymorphisms might potentially be protective factors or risk factors in specific leukemia types or among particular ethnicities.
